# l-β-aminoisobutyric acid, L-BAIBA, a marker of bone mineral density and body mass index, and D-BAIBA of physical performance and age

**DOI:** 10.1038/s41598-023-44249-6

**Published:** 2023-10-11

**Authors:** Charalampos Lyssikatos, Zhiying Wang, Ziyue Liu, Stuart J. Warden, Marco Brotto, Lynda Bonewald

**Affiliations:** 1 Indiana Center for Musculoskeletal Health, ICMH, Indiana University School of Medicine, VanNuys Medical Science Bldg, MS 5067A 635 Barnhill Drive, Indianapolis, IN 46202 USA; 2 Bone-Muscle Research Center, College of Nursing and Health Innovation, University of Texas-Arlington, Arlington, TX USA; 3 Department of Biostatistics and Health Data Science, Indiana University School of Medicine, Indianapolis, IN USA; 4 Department of Physical Therapy, School of Health and Human Sciences, Indiana University, Indianapolis, IN USA

**Keywords:** Physiology, Medical research

## Abstract

As both L- and D-BAIBA are increased with exercise, we sought to determine if circulating levels would be associated with physical performance. Serum levels of L- and D-BAIBA were quantified in 120 individuals (50% female) aged 20–85 years and categorized as either a “low” (LP), “average” (AP) or “high” performing (HP). Association analysis was performed using Spearman (S) and Pearson (P) correlation. Using Spearman correlation, L-BAIBA positively associated with (1) body mass index BMI (0.23) and total fat mass (0.19) in the 120 participants, (2) total fat mass in the 60 males (0.26), and (3) bone mineral density, BMD, (0.28) in addition to BMI (0.26) in the 60 females. In HP females, L-BAIBA positively associated with BMD (0.50) and lean mass (0.47). D-BAIBA was positively associated with (1) age (P 0.20) in the 120 participants, (2) age (P 0.49) in the LP females and (3) with gait speed (S 0.20) in the 120 participants. However, in HP males, this enantiomer had a negative association with appendicular lean/height (S − 0.52) and in the AP males a negative correlation with BMD (S − 0.47). No associations were observed in HP or AP females, whereas, in LP females, a positive association was observed with grip strength (S 0.45), but a negative with BMD (P − 0.52, S − 0.63) and chair stands (P − 0.47, S − 0.51). L-BAIBA may play a role in BMI and BMD in females, not males, whereas D-BAIBA may be a marker for aging and physical performance. The association of L-BAIBA with BMI and fat mass may reveal novel, not previously described functions for this enantiomer.

## Introduction

β-aminoisobutyric acid (BAIBA) is an aminobutyric acid (ABA) first discovered in 1951^1^. BAIBA has been shown to be involved in various metabolic processes such as the browning of white fat and hepatic β-oxidation^2^, improvement of glucose homeostasis through reducing insulin resistance in skeletal muscle^3^, prevention of diet induced obesity^4^, and protection against metabolic disturbance in type 2 diabetes^5^.

Plasma BAIBA levels are higher in young subjects than the elderly^6^ potentially because BAIBA expression is regulated by PGC-1α, which is higher in young compared to elderly individuals^7,8^. There is consensus that exercise increases circulating BAIBA. High plasma BAIBA concentrations were observed in humans undergoing aerobic exercise and were inversely correlated with metabolic risk factors, suggesting that BAIBA may protect against metabolic diseases^3,9–12^. Physical inactivity in patients on hemodialysis has been found to reduce plasma BAIBA concentrations^13,14^. In a study in obese and normal weight Native American boys and girls (11–17 years), blood BAIBA levels were measured after 16 weeks of aerobic exercise training and showed that the normal weight group had 29% higher BAIBA levels than those in the obese group suggesting an interaction with metabolic status^15^. Similar to findings in humans, total BAIBA is increased almost three fold in rats following 8 weeks of treadmill running compared to non exercised controls^16^.

However, what has not been taken into account with the studies referenced above is that BAIBA is produced as two enantiomers that have potentially different functions^17^. Only one study has measured both enantiomers in serum in response to exercise^11^. These investigators found that both enantiomers were increased in response to a single bout of exercise in recreationally active participants, supporting a mechanistic role for endogenous BAIBA in mediating the acute beneficial effects of exercise^11^. They found that D-BAIBA was 67 times higher than L-BAIBA at baseline in young subjects and that aerobic exercise produced a 13% increase in D-BAIBA and a 20% increase in L-BAIBA. These authors make the case for not treating BAIBA as one molecule. We had made this case previously based on our studies in mice where clear differences in potency were observed^17^ and make that case again here in the present study.

The two enantiomers of BAIBA are generated by different metabolic pathways^2,18,19^. D-BAIBA is catabolized from thymine that is endogenously synthesized and L-BAIBA from the essential branch chain amino acid valine, which is not endogenously synthesized and must be obtained from diet. They are also present in different organs; L-BAIBA in the brain, kidney, liver and muscle mitochondria and D-BAIBA in the liver and kidney^2,18,19^. We have previously shown that L- but not D-BAIBA is produced by murine contracting muscle^17^. Our studies in mice have shown that the L-BAIBA is 100 to 1000 times more potent than the D*-*BAIBA enantiomer in preventing osteocyte apoptosis, the first study to suggest that the enantiomers may have different potencies. The L form reduces bone and muscle loss due to hindlimb unloading in mice^17^ and will synergize with suboptimal loading to initiate new bone formation^20^.

To date, few studies in humans have investigated the role of the BAIBA enantiomers with regards to physical performance and musculoskeletal health. We had shown previously that D-BAIBA associated with physical activity in young lean women, 21–41 years, and with hip BMD in older women 48–80 years without osteoporosis/osteopenia^21^.

The aim of the present study was to determine if either of the two BAIBA enantiomers were associated with and could potentially serve as a biomarker for physical performance and physical parameters such as bone mineral density, BMD, BMI, total fat mass, and total lean mass in healthy individuals. The results provide clues regarding the different functions of the two enantiomers.

## Methods

### Recruitment of human subjects

The current cross-sectional study used serum samples and data retrieved for 120 individuals who had visited the Musculoskeletal Function, Imaging, and Tissue Resource Core (FIT Core) of the Indiana Center for Musculoskeletal Health’s Clinical Research Center (Indianapolis, Indiana) between 3/2018 and 4/2019. The FIT Core serves to provide: (1) standardized performance of physical function tests and patient reported outcomes related to physical function, (2) imaging outcomes for body composition and bone health, and (3) the collection and banking of biological samples within the Indiana Biobank.

Participants were recruited to the FIT Core by self-referral from the local community and by investigators seeking outcomes related to musculoskeletal health for their research subjects. The Core has Institutional Review Board approval from Indiana University (IU–IRB) for sample and data collection and storage from all-comers who provide written informed consent. The consent provided by participants allows for deidentified samples and data to be stored and retrieved. Additional approval was obtained from the IU-IRB for the current analyses. All methods were performed in accordance with the Declaration of Helsinki and were approved by the IU-IRB (IU-IRB #1707550885).

The FIT Core collected samples and data from 1518 individuals between 3/2018 and 4/2019. To be included in the current analyses, individuals needed to be 20–85 years of age, self-reported white and non-Hispanic, and without a self-reported major chronic disease. The lower age limit of 20 years was selected to exclude the potential impact of growth. The upper age limit of 80 yrs was selected because the FIT Core cohort currently has a limited number of participants beyond this age. Analyses were restricted to white and non-Hispanic individuals to promote homogeneity within the study population. Race and ethnicity are important variables, but were not the topic of investigation in the current study.

**Figure 1:**
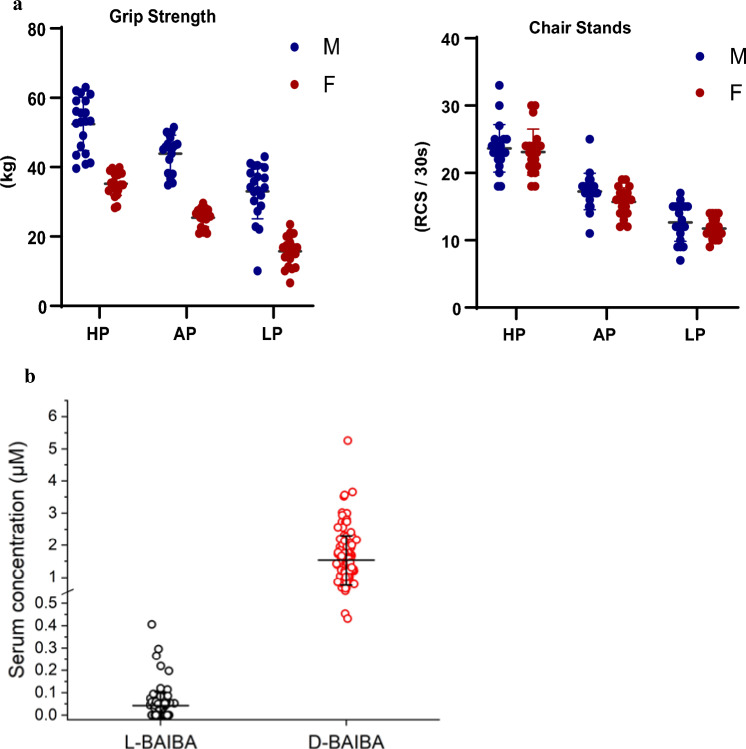
(**a**) Classification of performing groups—high [HP], average [AP], low [LP]. Participants were ranked for their performance on best grip strength and number of repeat chair stands completed in 30 s. (**b**) Comparison of serum *L*-BAIBA and D-BAIBA levels. Comparison of serum *L*-BAIBA levels from the groups containing all participants (n = 120) and only with L-BAIBA detectable (n = 69). Mean ± SD. *L-BAIBA was detected from 69 out of 120 participants. ^1^For the participants with L-BAIBA undetectable in their serum samples, the L-BAIBA levels are counted as "0".

Individuals within each sex were stratified into four age groups (20–34, 35–49, 50–64, and 65+ yrs) and ranked for their performance on the FIT Core’s hand grip strength test and test of the number of chair stands completed in 30 s (Fig. 1a). These tests were selected as they assess function of the upper and lower extremities, are predictive of poor outcomes, and are the two skeletal muscle strength tests recommended for identifying sarcopenia^22^. The age-and sex-specific rankings on each of the two tests were summed within each individual to create a single composite ranking. The 5 individuals within each sex and age range with the lowest, average, and highest composite rank were selected and grouped as low (LP), average (AP), and high (HP) performers, respectively. The categorization into groups ensured inclusion of individuals across the entire ranges of performances (supplementary Tables S1 and S2).

### Physical function

The FIT Core assessed dominant hand grip strength (Jamar Plus+ digital hand dynamometer; Sammons Preston, Bolingbrook, IL), the number of chair stands completed in 30 s, and the time taken to complete 5 chair stands, as we have previously described^23^. In addition to raw values, grip strength and repeat chair stand outcomes were converted to age- and sex-matched *z* scores relative to reference data obtained in the FIT Core^23^. Time to walk 4-m from a stationary start at normal speed (usual gait speed) and as quickly as possible without running (fast gait speed) were measured with a stopwatch and converted to speed (m/s), as we have previously reported^24^.

Results from the repeat chair stands, usual gait speed, and a static balance test (ability balance for 10 s with feet side-by-side, semi-tandem, and tandem) were used to calculate the Short Physical Performance Battery (SPPB) score out of 12^25^. Distance walked in 6 min was measured according to the American Thoracic Society^26^ on a 20-m long indoor course. The physical function (PF) domain of the NIH Patient Reported Outcomes Measurement Information System (PROMIS) computerized adaptive test (CAT) (PROMIS-CAT-PF) (version 1.2) and the physical functioning subscale of the Short Form 36 (SF-36 PF) were used to assess self-reported functional health.

### Body composition and bone health

Participant height (to nearest 0.1 cm) and mass (to nearest 0.1 kg) were measured without shoes using a calibrated stadiometer (Seca 264; Seca GmbH & Co., Hamburg, Germany) and scale (MS140-300; Brecknell, Fairmont, MN), respectively. Body mass index (BMI; kg/m^2^) was calculated as body mass relative to height squared. Appendicular lean mass relative to height squared (kg/m^2^) and whole-body aBMD, fat mass, percent were assessed by whole-body dual-energy x-ray absorptiometry (DXA) (Norland Elite; Norland at Swissray, Fort Atkinson, WI). Regional DXA using the same scanner assessed hip and spine aBMD.

### Chemicals and reagents

Aminobutyric acid standard compounds (S)-3-aminoisobutyric acid (*L*-BAIBA) and (R)-3-aminoisobutyric acid (*D*-BAIBA) were purchased from Adipogen Corp. (San Diego, CA). G. Isotopic internal standard (IS) compounds (±)-3-amino-iso-butyric-2,3,3-d_3_ acid (*D,L*-BAIBA-d_3_) were obtained from CDN Isotopes (Pointe-Claire, Quebec, Canada). Formic acid (reagent grade, ≥ 95%), Bovine Serum Albumin (BSA) were obtained from Sigma–Aldrich (St. Louis, MO). Phosphate Buffered Saline (PBS) was purchased from Fisher Scientific (Pittsburgh, PA). HPLC–MS grade acetonitrile, water, and methanol were purchased from J.T. Baker (Phillipsburg, NJ).

### LC–MS/MS conditions

All components of liquid chromatography-tandem mass spectrometry (LC–MS/MS) system are from Shimadzu Scientific Instruments, Inc. (Columbia, MD). The LC system was equipped with pumps A and B (LC-30AD), and autosampler (SIL-30AC). The LC separation was conducted on a chiral SPP-TeicoShell column (150 × 4.6 mm, 2.7 µm, AZYP LLC., Arlington, TX) configured with a Synergi™ 4 µm Max-RP column as guard column (50 × 2.0 mm, Phenomenex, Torrance, CA). The MS/MS analysis was performed on Shimadzu LCMS-8050 triple quadrupole mass spectrometer.

Quantification of isomeric aminobutyric acids in human serum samples was followed the LC–MS/MS method as published previously^21^. Briefly, mobile phases are methanol (A) and water containing 0.005% formic acid and 2.5 mM ammonium formate (B). The MS instrument was operated and optimized under positive electrospray (+ESI) and multiple reaction monitoring modes (MRM). The *m/z* transitions (precursor to product ions) and their tuning voltages were selected from published paper^21^ and further optimized based on the best MRM responses from instrumental method optimization software. All analyses and data processing were completed on Shimadzu LabSolutions V5.91 software (Shimadzu Scientific Instruments, Inc., Columbia, MD).

### Sample preparation for LC–MS/MS analysis

Ten microliter human serum samples and same volume of IS mixture solution (1.2 µM, 0.1% formic acid in methanol, v/v) were added to 35 µL 0.1% (v/v) formic acid in methanol, followed by 20 min-shaking at room temperature and another 15 min-centrifugation at 15,000×*g*, 4 °C to precipitate the proteins. The supernatant was directly transferred to autosampler vial and 45 μL of each sample was injected for LC–MS/MS analysis.

The samples of standard calibration curves were prepared by spiking the pure standards in surrogate matrix 5% (w/v) BSA in PBS (pH7.4). The samples for ten-point calibration curves were prepared by diluting the working solution to 0.02–10.24 µM for L-BAIBA and D-BAIBA. Then ten microliters of each standard sample were taken and treated following the same preparation procedures of serum samples for LC–MS/MS analysis. These are the same samples as used in the sister manuscript in this issue^27^.

### Statistical analysis

Data were summarized as mean ± SD. Comparisons among groups were performed using Student’s t-test and one-way ANOVA with Tukey’s post-Hoc test (α = 0.05). Association analysis was performed using both Pearson (P) correlations and Spearman (S) correlations. To control for the effects of age and BMI, partial correlations were further calculated. SAS 9.4 (SAS Institute, Cary, NC, USA) was used for statistical analysis. Two-sided p-values < 0.05 were considered as significant. Both Pearson (P) and Spearman (S) correlations were examined for associations with D-BAIBA to characterize these associations more extensively than a single correlation type, while Spearman correlation is more robust to outliers and able to capture nonlinear but monotonic associations (Pearson correlation captures the linear ones better). For this reason, for L-BAIBA statistical analysis only the Spearman correlations were used. For those with L-BAIBA undetectable in their serum samples (some L-BAIBA levels were under the limit of detection and their values could not be determined) the L-BAIBA levels are counted as "0". Heatmaps were generated to display correlations with the magnitude coded by colors and numbers been displayed for the ones with p value < 0.05. With N = 120 as the total sample, N = 60 for female and male separately, and N = 20 for each sex/function combination, we had an 80% power at type I error level 0.05 to detect Pearson and Spearman correlations as 0.25 and 0.27, 0.35 and 0.37, and 0.58 and 0.61, respectively.

### Ethics approval and consent to participate

Written informed consent was obtained prior any study procedure according to the declaration of Helsinki and Indiana University Institutional Review Board (IU–IRB, study number 1707550885). After the protocol consent form has been signed and dated by each study participant, 20 mL of blood was collected. Specimens were then aliquoted, prepared for storage and frozen at − 80 °C until further analyses at the Indiana Biobank.

## Results

In this study, serum levels of L- and D-BAIBA were quantitated in 120 individuals aged 20–85 (characteristics Table 1), 60 women and 60 men classified as “low”, “average” or “high” performers, (n = 20) according to physical performance tests of best grip strength and repeat chair stands (RCS) (Fig. 1a).

**Table 1:** Characteristics of the 120 participants.

All	N	Mean	SD	Median	Min	Max
Age (years)	120	49.60	17.58	50.08	20.09	84.73
Height (cm)	120	170.70	8.91	169.55	149.00	191.80
Weight (kg)	120	78.13	13.62	77.80	54.10	114.10
BMI (kg/m^2^)	120	26.83	4.48	25.55	19.70	45.10
Appendicular lean/height^2^ (kg/m^2^)	117	7.84	1.39	7.98	4.64	12.14
Total BMD (g/cm^2^)	116	1.11	0.15	1.11	0.77	1.43
Spine BMD (g/cm^2^)	117	1.09	0.17	1.09	0.68	1.45
Femoral neck BMD (g/cm^2^)	117	0.89	0.17	0.85	0.55	1.35
Total SPPB score	120	11.56	1.38	12.00	0.00	12.00
SPPB gait speed score	120	3.99	0.09	4.00	3.00	4.00
Usual gait speed (m/s)	120	1.40	0.18	1.40	0.74	2.00
Fast gait speed (m/s)	120	2.05	0.34	2.05	1.01	3.28
Best grip strength (kg)	120	34.53	12.80	34.85	6.60	63.00
Time for 5 chair stands (s)	120	8.87	2.96	8.08	2.13	19.77
6-min walk distance (m)	117	569.62	109.81	580.00	160.00	969.00
PROMIS score	120	56.42	7.27	54.70	40.40	73.30
SF-36 PFS raw score	120	92.96	12.79	100.00	20.00	100.00

### Participant characteristics

There were no age group X function group interactions for any participant characteristics (all *p* = 0.06 to 0.34). Grip strength and chair stands completed in 30-s in both females and males were lower with higher age group and lower function group (all p ≤ 0.001). The average hand grip z-score in LP, AP, and HP groups across both sexes was − 1.22 (95% confidence [CI] − 1.42 to − 1.02), 0.10 (95% CI − 0.10 to 0.30), and 1.36 (95% CI 1.17 to 1.56), respectively. The average z-score for chair stands completed in 30-s in LP, AP, and HP groups across both sexes was − 1.15 (95% confidence [CI] − 1.32 to − 0.98), 0.07 (95% CI − 0.10 to 0.25), and 1.53 (95% CI 1.36 to 1.70), respectively.

The concentrations for D-BAIBA and L-BAIBA in the study population ranged from 1.53 ± 0.77 μM and 0.043 ± 0.0060 μM, respectively, approximately a 40-fold difference in expression (Fig. 1b).

There were no significant differences in L-BAIBA concentrations between the different physical performance function groups (LP: low performers, AP: average performers, HP performers) as summarized in Table 2. Similarly, no statistically significant difference was noted between the three physical performance groups with the four age groups (20–34, 35–49, 50–64 and older adults 65+) (Table 3) nor with gender (Table 4).

**Table 2:** Serum concentrations of L- and D-BAIBA in different physical performance populations.

Aminobutyric acids	HP	AP	LP	Overall
Sample size N	40	40	40	120
*L*-BAIBA (µM)	0.034 ± 0.030	0.054 ± 0.096	0.040 ± 0.030	0.043 ± 0.060
*D*-BAIBA (µM)	1.65 ± 0.94	1.46 ± 0.61	1.49 ± 0.72	1.53 ± 0.77

**Table 3:** Serum concentrations of L- and D-BAIBA in different age populations.

Aminobutyric acids	Measurements	Physical performance	Age group				
			20–34 yrs	35–49 yrs	50–64 yrs	65+ yrs	p-value
*D-*BAIBA (µM)	All participants in each group	All	1.38 ± 0.62 (30)	1.51 ± 0.65 (30)	1.47 ± 0.67 (30)	1.77 ± 1.03 (30)	0.2374
		High (HP)	1.54 ± 0.79 (10)	1.43 ± 0.40 (10)	1.56 ± 0.87 (10)	2.07 ± 1.41 (10)	0.4432
		Average (AP)	1.31 ± 0.56 (10)	1.63 ± 0.69 (10)	1.38 ± 0.66 (10)	1.51 ± 0.56 (10)	0.662
		Low (LP)	1.30 ± 0.53 (10)	1.48 ± 0.83 (10)	1.46 ± 0.48 (10)	1.73 ± 0.98 (10)	0.6314
*L-*BAIBA (µM)	All participants in each group^1^	All	0.025 ± 0.028 (30)	0.041 ± 0.044 (30)	0.052 ± 0.066 (30)	0.052 ± 0.085 (30)	0.2572
		High (HP)	0.037 ± 0.030 (10)	0.038 ± 0.036 (10)	0.029 ± 0.028 (10)	0.033 ± 0.030 (10)	0.9048
		Average (AP)	0.0052 ± 0.016 (10)	0.047 ± 0.064 (10)	0.081 ± 0.106 (10)	0.084 ± 0.140 (10)	0.2243
		Low (LP)	0.033 ± 0.027 (10)	0.038 ± 0.028 (10)	0.048 ± 0.027 (10)	0.039 ± 0.038 (10)	0.7553

For the participants with L-BAIBA undetectable in their serum samples, the L-BAIBA levels are counted as "0".

**Table 4:** Serum concentrations of L- and D-BAIBA in different gender populations.

Aminobutyric acids	Measurements	Physical performance	Gender		
			Female	Male	p-value
*D-*BAIBA (µM)	All participants in each group	All	1.55 ± 0.76 (60)	1.52 ± 0.78 (60)	0.8306
		High (HP)	1.58 ± 0.70 (20)	1.72 ± 1.14 (20)	0.6427
		Average (AP)	1.51 ± 0.71 (20)	1.40 ± 0.51 (20)	0.5449
		Low (LP)	1.55 ± 0.88 (20)	1.44 ± 0.53 (20)	0.6291
*L-*BAIBA (µM)	All participants in each group^1^	All	0.037 ± 0.064 (60)	0.048 ± 0.064 (60)	0.3348
		High (HP)	0.036 ± 0.031 (20)	0.033 ± 0.030 (20)	0.8138
		Average (AP)	0.036 ± 0.092 (20)	0.072 ± 0.098 (20)	0.2421
		Low (LP)	0.040 ± 0.024 (20)	0.039 ± 0.035 (20)	0.8848

Each data represents mean ± SD (n). ANOVA was applied for statistics analysis. *p < 0.05.

For the participants with *L*-BAIBA undetectable in their serum samples, the* L*-BAIBA levels are counted as "0".

### Correlations of physical performance and physical parameters in all 120 samples, 60 females and 60 males, age 20–85

**Figure 2:**
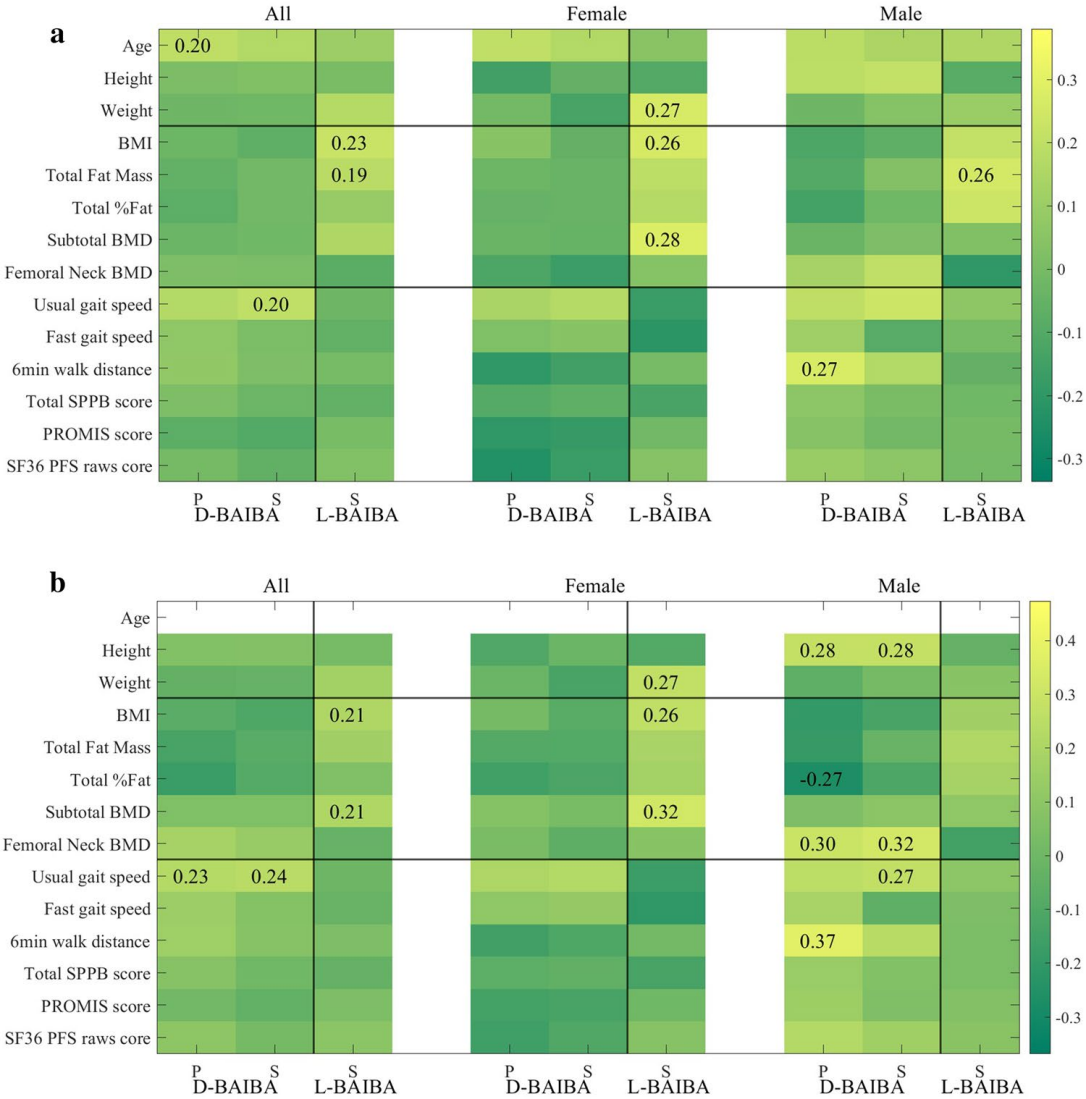
(**a**) Heatmap of D- and L- BAIBA with physical performance and physical parameters. In all 120 samples, 60 females and 60 males, age 20–85. (**b**) After the age effect was removed (P—Pearson and S—Spearman).

When all 120 samples (60 females and 60 males, age 20–85) were examined, L-BAIBA had a positive Spearman association with BMI (0.23, p < 0.05) and with total fat mass (0.19, p < 0.05) (Fig. 2a and supplementary Table S3b). D-BAIBA had a positive Pearson (P) correlation with age (0.20, p < 0.05) and positive Spearman (S) association with usual gait speed (0.20, p < 0.05) (Fig. 2a and supplementary Table S3a). No significant correlations were observed with any other parameters.

When the 120 participants were divided between females and males, in the 60 female participants, L-BAIBA had a positive Spearman association with BMI (0.26, p < 0.05), weight (0.27, p < 0.05) and subtotal BMD (0.28, p < 0.05) (Fig. 2a and supplementary Table S4a). L-BAIBA in the 60 males was positively related with total fat mass (0.26, p < 0.05) (Fig. 2a and supplementary Table 4b). D-BAIBA was positively correlated with 6MWT (0.27, p < 0.05) in the 60 males. No significant correlations were observed with any other parameters.

With the age effect removed most of the above correlations remained significant; especially the positive corrrelations of L-BAIBA with weight, BMI, and subtotal BMD in females and BMI in the total population. The positive correlation of D-BAIBA with gait speed in the total population and 6MWT in males was retained. However, the correlation of L-BAIBA with total fat mass in the total population was lost, but a correlation with subtotal BMD was gained. This indicates that these two parameters are not age associated. With the effects of age removed, several new correlations of D-BAIBA was observed in males. D-BAIBA in males gained a positive correlation with height, (P 0.28 and S 0.28), positive P (0.30) and S (0.32) correlation with femoral neck BMD, retained a positive S (0.27) with usual gait speed, but gained a positive correlation with 6MWT (P 0.37) and gained a negative correlation with total %fat (P − 0.27) (Fig. 2b and supplementary Table S5). A positive D-BAIBA association with femoral neck BMD in males was seen after the age and BMI effects (P 0.32, p < 0.05, S 0.33, p < 0.05) were removed (supplementary Fig. 1a and supplementary Tables S7 and S8).

The correlation with gait speed was lost when divided between males and females but D-BAIBA in males had a positive Pearson correlation (0.27, p < 0.05) with the 6MWT (Fig. 2a).

### Correlations of physical performance and physical parameters in HP, AP and LP groups

#### High performing (HP) individuals

**Figure 3:**
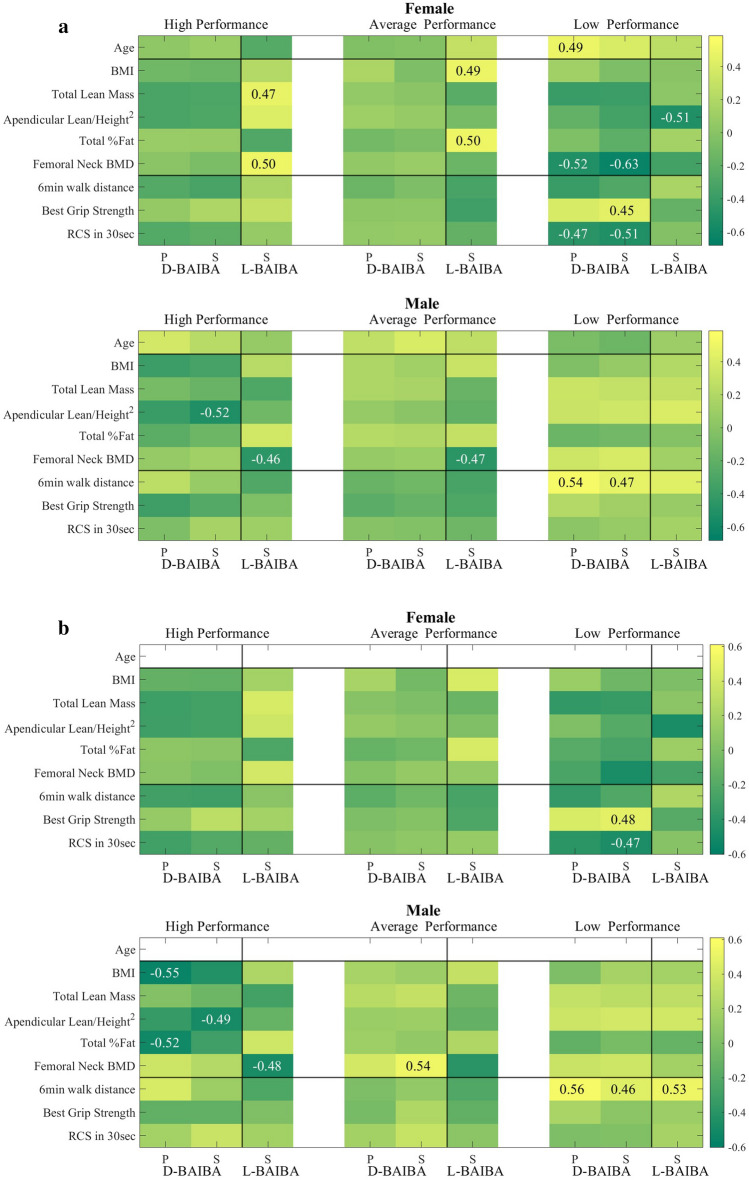
(**a**) Heatmap of D- and L-BAIBA with characteristics of physical performance, BMI and BMD. in HP, AP and LP in females and males. (**b**) After the age effect was removed (P—Pearson and S—Spearman).

In females L-BAIBA had a positive Spearman correlation with femoral neck BMD (S 0.50, p < 0.05) and total lean mass (0.47, p < 0.05), but did not remain significant after age effect was removed (Fig. 3a,b). This positive correlation with femoral neck BMD in females was opposite from what was found in HP males; and remained significant after age and BMI effects had been removed (0.48, p < 0.05, supplementary Fig. 1b). Also, there was a negative L-BAIBA association with the total fat percent, after age and BMI effects were removed (− 0.53, p < 0.05) (supplementary Fig. 1b and supplementary Table S4a). No significant correlations were observed with any other parameters.

In the group of HP males, L-BAIBA had a negative association with femoral neck BMD (S − 0.46, p < 0.05) and D-BAIBA had a negative association (S − 0.52, p < 0.05) with appendicular lean/height^2^ (kg/m^2^) (Fig. 3a and supplementary Table S5). Both these associations remained significant after age effect had been removed (− 0.48 and − 0.49, respectively) (Fig. 3b) suggesting that L-BAIBA is associated with BMD and D-BAIBA with appendicular height regardless of age.

#### Average performing (AP) individuals

In females, L-BAIBA had a positive association with BMI (S 0.49, p < 0.05) and total fat percent (0.50, p < 0.05) (Fig. 3a and supplementary Table S4a), and in males, L-BAIBA had a negative association with the femoral neck BMD (− 0.47, p < 0.05) (Fig. 3a and supplementary Table S4b). These were lost when age was accounted for suggesting this is more of an age association.

The only association observed with D-BAIBA was femoral neck BMD when age was accounted for. This suggests that D-BAIBA is associated with BMD and age in this group.

#### Low performing (LP) individuals

The correlation of D-BAIBA with age as shown in Fig. 2a for all 120 participants was lost in all of the performance groups except in the female LP group. This group may be responsible for the significant correlations observed with all 120 participants.

In the LP females, there was a negative association of L-BAIBA with appendicular lean/height^2^ (kg/m^2^) (− 0.51, p < 0.05) (Fig. 3a), that no longer associated when age was removed (Fig. 3b) but remained significant after age plus BMI effects had been removed (− 0.52, p < 0.05) suggesting that BMI might be responsible for this feature in LP females (supplementary Fig. 1 and supplementary Table S4a).

L-BAIBA in the LP male group had a positive association with the 6MWT (S 0.53, p < 0.03) after age effect had been removed (Fig. 3b and supplementary Table S4b). No significant correlations were observed with any other parameters.

In females, D-BAIBA had a positive correlation with age (P 0.49, p < 0.05), positive correlation with best grip strength (S 0.45, p < 0.05), negative associations by both Pearson and Spearman with the RCS in 30 s (P − 0.47, p < 0.05 and S − 0.51, p < 0.05), and femoral neck BMD (P − 0.52, p < 0.05, S − 0.63, p < 0.05) (Fig. 3a**)**. The Spearman correlations for best grip strength (S 0.48, p < 0.05) and RCS in 30 s (S − 0.47, p < 0.05) remained significant after age effect had been removed (Fig. 3b and supplementary Table S6). Detailed D-BAIBA correlations in LP females with physical performance parameters are shown in the supplementary Tables S9 and S10.

D-BAIBA in LP males had a positive correlation with 6MWT (P 0.54, p < 0.05 and S 0.47, p < 0.05) (Fig. 3a). This correlation remained significant after the age effect had been removed (P 0.56, p < 0.05, and S 0.46, p < 0.05) (Fig. 3b, supplementary Tables S7 and S8).

## Discussion

Muscle and bone communicate through the production of myokines and osteokines^28^. During exercise, contracted muscle and loaded bone produce beneficial signaling molecules while unloaded, sedentary muscle and unloaded, resorbing bone produces molecules that have negative effects on the opposing tissue. Myokines can have autocrine, paracrine, and endocrine functions^29^. An example of a negative myokine is myostatin that negatively regulates skeletal and cardiac muscle, and reduces bone mass^30,31^. An example of a positive myokine is irisin^32^, that like BAIBA is associated with browning of white adipose tissue and with energy metabolism^9^ but also associated with brain health^33^. We found that murine contracted muscle produces L-BAIBA^17^, but the source of elevated D-BAIBA with exercise is unknown. Here we focus on the contracted muscle metabolite, L-BAIBA and its enantiomer D-BAIBA both known to be elevated with exercise in humans^11^ [To obtain a more comprehensive list of factors produced by sedentary and contracted muscle see^28,34^].

We had shown previously that D-BAIBA associates with physical activity in young lean women, 21–41 years old, and with hip BMD in older women, 48–80 years old, without osteoporosis or osteopenia^21^. In the present study, we found an association of D-BAIBA with physical performance. The main difference in these two studies was that questionnaires were used to determine physical activity in the Wang et al. study^21^, while the present study used a combination of questionnaires and actual physical performance measurements. Combining the results of both studies provides strong support that D-BAIBA is a marker of physical activity and performance. However, the positive association of L-BAIBA with BMI in females and fat mass in males came as a surprise as it has been shown that BAIBA induces conversion of white to brown fat^9^. A recent study has shown that plasma BAIBA concentration in heart failure patients is inversely correlated with indexes of fat mass, indicating that BAIBA may be a therapeutic target for excessive fat accumulation, but these investigators were measuring total BAIBA^35^. In another study, a low calorie diet increased BAIBA in relation to reduced body weight and pancreatic function in women with obesity^36^. These observations were based on measurements of total BAIBA which includes both enantiomers and/or racemic mixtures of BAIBA^35,36^. As D-BAIBA is 40–60 times greater in human serum than L-BAIBA, this particular function of reducing fat mass is most likely due to D-BAIBA.

Papers and reviews have been written assuming that L- and D-BAIBA have essentially the same functions^13,36–38^. We and others have emphasized that this assumption should not be made^17^. Within the past year, one publication has begun to note an association of L-BAIBA with fat metabolism. An association of an antipsychotic, clozapine, that induces an increase in fat mass with the production of L-BAIBA in the hypothalamus was observed^39^. This is the first publication to show an association between increased fat mass and L-BAIBA. In the present study, this association of fat mass with L-BAIBA may be reversed or nullified with exercise as the high performing females had an association of L-BAIBA with lean mass and not BMI.

For this retrospective single center study, our hypothesis was that physical performance would correlate with serum levels of the BAIBA enantiomers and therefore these would serve as biomarkers for high or low physical performance. The primary goal was to determine if a correlation existed between L- or D- BAIBA in both males and females from different ages (20–34, 35–49, 50–64, 65+) and with different physical tests to generate different levels of physical performance (LP, AP and HP groups). Our hypothesis was based on several studies showing an effect of exercise on BAIBA. A study by Roberts et al. performed with 80 sedentary but healthy participants, both genders, mean age of 34 years, with a 20-week aerobic training showed a total BAIBA increase of 17%^9^. A 2016 study in 49 patients with chronic kidney disease on hemodialysis, showed that BAIBA was lower in physically inactive patients compared to active patients (able to exercise at least once a week)^13^. In 2017 a cyclic exercise study with 13 untrained male participants showed a small increase in total BAIBA during exercise only at 4 h^38^. The first clinical study where the enantiomers and not total BAIBA were quantitated was in 2019 and showed that L-BAIBA increased during aerobic exercise and continued to increase at 30 min after participants completed the exercise schedule. D-BAIBA also increased during exercise but dropped at 30 min after the exercise schedule^11^. Therefore, we assumed that a positive correlation of both enantiomers would be observed in high performing participants as it was assumed that these individuals exercise more frequently than the low performing participants. However, data analysis of the 120 human serum samples, did not support the hypothesis of elevated D or L-BAIBA in high performing individuals.

In the present study, for the 120 total participants, L-BAIBA positively correlated with BMI and total fat mass. In the 60 male participants, L-BAIBA associated with total fat mass in contrast to the 60 female participants, where L-BAIBA correlated with BMI and with subtotal and total BMD. One must keep in mind that BMI is a rough estimate of relative body weight, and is not accurate for body composition and does not include fat mass^40^. With regards to physical parameters in the different physical performance groups, in the male high performing group, L-BAIBA was negatively linked with femoral neck BMD whereas in the female high performing group, L-BAIBA had a positive association with femoral neck BMD and lean mass. This data suggests sex-specific effects and/or function for L-BAIBA. In the average male performers, L-BAIBA continued to have a negative association with femoral neck BMD, while in females, L-BAIBA had a positive association with BMI and total % fat. In low performing males L-BAIBA had a positive association with 6MWT, and in low performing females a negative association with appendicular lean/height^2 (kg/m^2). It was surprising that L-BAIBA would be so strongly associated with parameters of fat mass. However, in the female high physical performance group, this was reversed and became associated for lean mass, not BMI. This suggests that exercise which reduces fat mass and increases lean mass along with increased BMD may determine the function of L-BAIBA. Our recent in vivo murine studies show that L-BAIBA enhances the effects of suboptimal loading of bone^20^, which supports the concept that L-BAIBA, elevated with exercise, may enhance the positive effects of exercise on bone.

For the 120 total participants, D-BAIBA positively associated with age and gait speed. In the 60 male participants, D-BAIBA positively associated with femoral neck BMD and six-minute walk test (6MWT). With regards to physical performance, in the male high performing group, D-BAIBA had a negative correlation with appendicular lean/height^2 (kg/m^2). In the low performing males, D-BAIBA had a positive correlation with 6MWT and with age. It is not clear why this enantiomer would be associated with age. Also, it is not clear why D-BAIBA would be positively associated with the 6MWT. Additional studies will be required.

There are several limitations to our study. Although the chosen samples for analysis were from study participants without chronic disease—not on prescription medications (for instance not on statins which are known to be associated with myopathy); there are some limitations. Any lack of significance could be due to the retrospective nature of the study from already collected blood samples, the fact that this is a single center study and the relatively small cohort of 120 samples may account for the lack of significance. Another limitation is not knowing how soon after exercise the blood was drawn as it has recently been described that BAIBA secretion increases during and after exercise^41^. Other confounding factors that could be considered include circadian rhythm, diurnal variation/time of the day in which blood samples were collected, the fact that the blood draws were either at the beginning or the end of the physical performance tests, exercise status is unknown, as is the use of vitamins and /or supplements. Hormone replacement or use of contraception was unknown in the samples collected from female participants. Diet was not controlled before participating in the study and dietary history was not collected. Also, the conversion of D-BAIBA to L-BAIBA and vice versa during exercise cannot be excluded. A future study where the above factors can be controlled, could provide more information regarding the release, timing, and response of D and L-BAIBA to exercise.

## Conclusions

In summary, L-BAIBA did not show major associations with physical performance except for 6MWT only in low performing males, but did show significant associations with the physical characteristics of BMI, fat mass, lean mass, and femoral neck BMD, while D-BAIBA showed significant associations with age and physical performance (usual gait speed, 6MWT, grip strength, RCS) features. There were clearly sex specific differences in associations of L-BAIBA with BMD, BMI, and fat mass. The mechanisms responsible for these associations remain to be explored but suggest differences in function for the two enantiomers.

### Supplementary Information

Supplementary Information.

## Data Availability

All data generated and analyzed during this study are included in this published article and its supplementary information files.

## Abbreviations

6MWT: Six-minute walk test
ABAs: Aminobutyric acids
BAIBA: β-Aminoisobutyric acid
BMD: Bone mineral density
BMI: Body mass index
BSA: Bovine serum albumin
ESI: Electrospray Ionization
IS: Internal standard
LC: Liquid chromatography
LC–MS/MS: Liquid chromatography-tandem mass spectrometry
MRM: Multiple reaction monitoring
MS: Mass spectrometry
m/z: Mass-to-charge ratio
PBS: Phosphate-buffered saline
MSK: Musculoskeletal
SD: Standard deviation
