# Supplementary material for: l-β-aminoisobutyric acid, L-BAIBA, a marker of bone mineral density and body mass index, and D-BAIBA of physical performance and age
Source: Sci Rep. 2023 Oct 11;13:17212. doi: 10.1038/s41598-023-44249-6 (PMC10567793; doi:10.1038/s41598-023-44249-6)
Supplement: Supplementary file 1 — Supplementary Information. [file 41598_2023_44249_MOESM1_ESM.pdf]

L-β-aminoisobutyric acid, L-BAIBA, a marker of bone mineral density and body mass index, and D-BAIBA of physical performance and age.

Authors:

Charalampos Lyssikatos. MD[1,┼], Zhiying Wang PhD[2,┼], Ziyue Liu PhD, BMED[1,3], Stuart J. Warden PT, PhD, FACSM, FASBMR[1,4], Marco Brotto, BSN, MPharm, PhD[2], Lynda Bonewald PhD[1*]

[1]Indiana Center for Musculoskeletal Health, Indiana University School of Medicine, Indianapolis, IN, USA

[2]Bone-Muscle Research Center, College of Nursing and Health Innovation, University of Texas-Arlington, Arlington, TX, USA

[3]Department of Biostatistics and Health Data Science, Indiana University School of Medicine, Indianapolis, IN, USA

[4]Department of Physical Therapy, School of Health and Human Sciences, Indiana University, Indianapolis, IN, USA

Supplemental figure 1. Heatmap of D- and L- BAIBA after the age and BMI effect was removed in **a.** all 120 samples, 60 females and 60 males, age 20- 85. and **b.** in the subgroups (LP, AP, HP)

a.

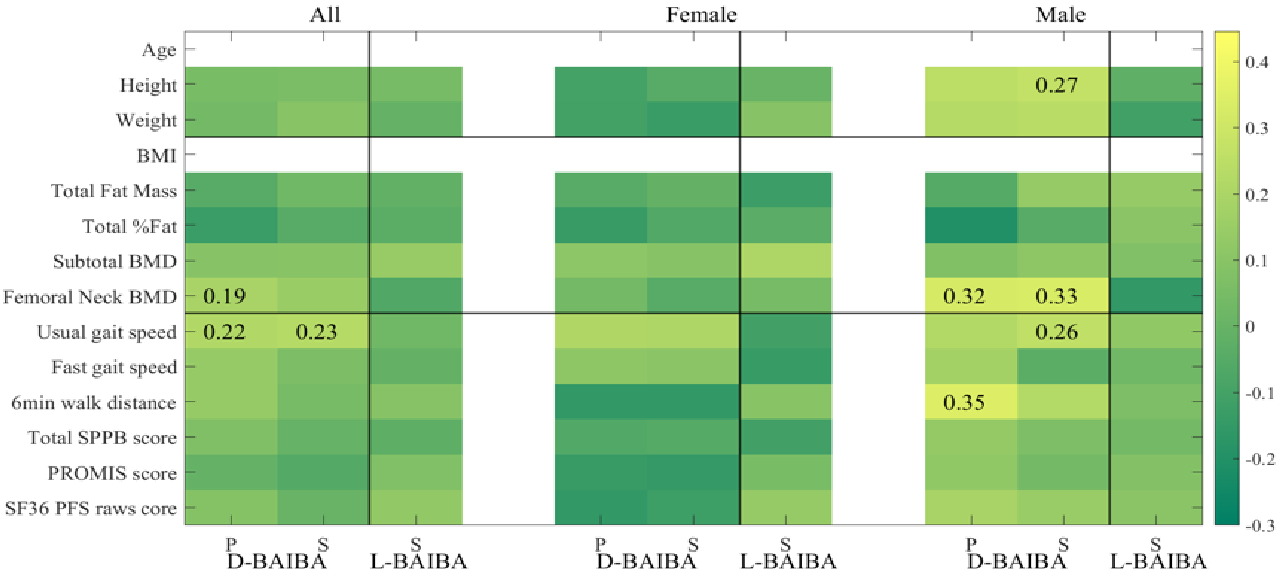
All
Female
Male
Age
Height
Weight
BMI
Total Fat Mass
Total %Fat
Subtotal BMD
Femoral Neck BMD
Usual gait speed
Fast gait speed
6min walk distance
Total SPPB score
PROMIS score
SF36 PFS raws core
0.27
0.19
0.32
0.33
0.22
0.23
0.26
0.35
P
S
D-BAIBA
L-BAIBA
0.4
0.3
0.2
0.1
0
-0.1
-0.2
-0.3

b.

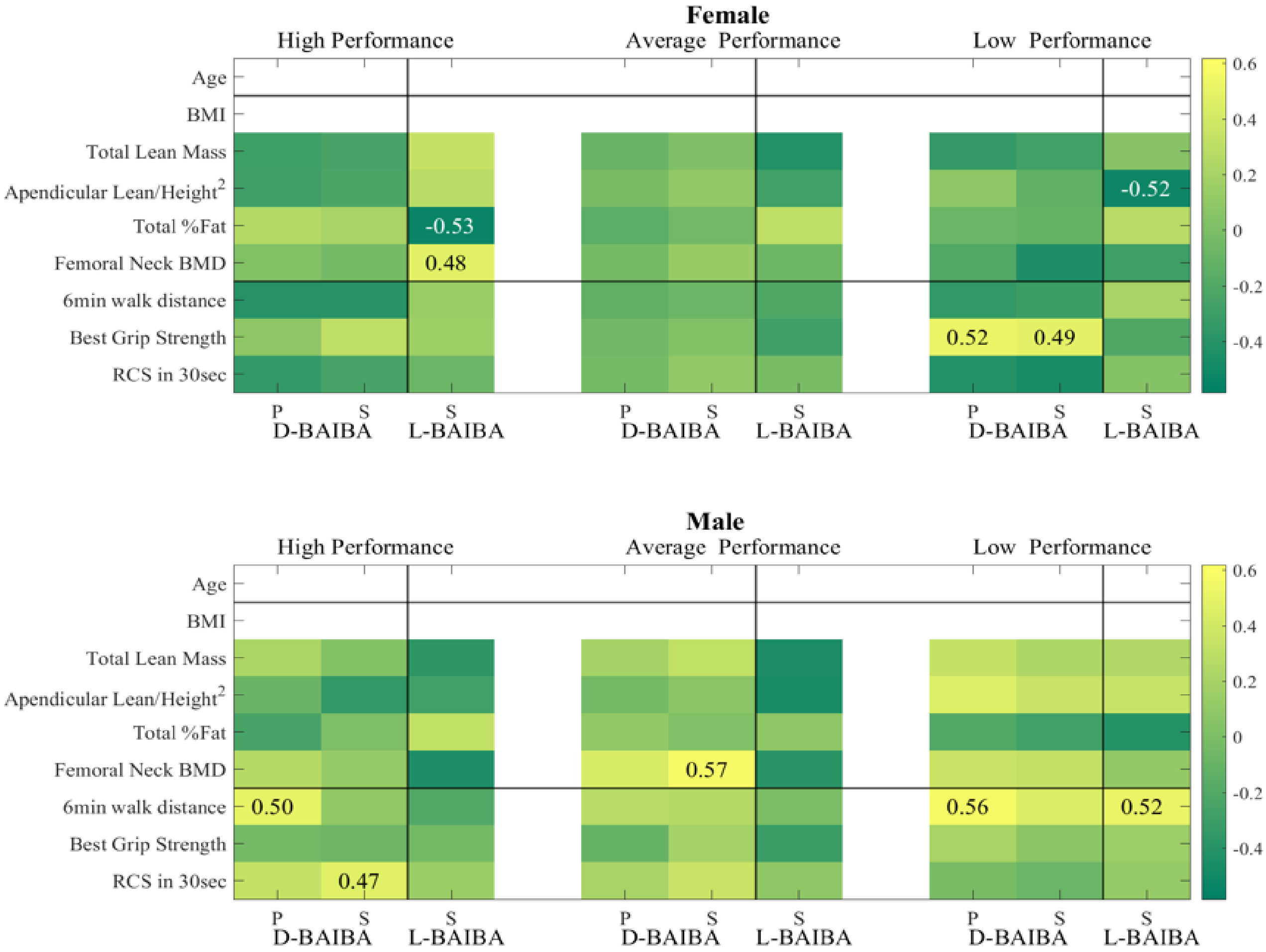
Female
High Performance
Average Performance
Low Performance
Age
BMI
Total Lean Mass
Apendicular Lean/Height²
Total %Fat
Femoral Neck BMD
6min walk distance
Best Grip Strength
RCS in 30sec
-0.53
0.48
-0.52
0.52
0.49
P
S
D-BAIBA
L-BAIBA
0.6
0.4
0.2
0
-0.2
-0.4
Male
High Performance
Average Performance
Low Performance
0.57
0.50
0.56
0.52
0.47

Supplementary tables:

Table S1. Characteristics on separate groups by performance (low, average, high).

	FEMALES											
	20-35 years			35-50 years			50-65 years			65+		
	Low	Average	High	Low	Average	High	Low	Average	High	Low	Average	High
Age (yr)	26.6 ± 3.8	27.4 ± 4.2	27.5 ± 5.2	44.3 ± 5.5	45.2 ± 3.5	45.1 ± 5.1	57.2 ± 4.4	57.9 ± 2.7	53.3 ± 1.3	76.4 ± 7.1	73.9 ± 1.6	68.3 ± 3.5
Height (m)	1.62 ± 0.07	1.68 ± 0.05	1.67 ± 0.05	1.63 ± 0.05	1.65 ± 0.06	1.69 ± 0.05	1.63 ± 0.02	1.64 ± 0.06	1.69 ± 0.08	1.62 ± 0.08	1.59 ± 0.04	1.64 ± 0.04
Weight (kg)	67.4 ± 10.3	63.1 ± 5.2	69.0 ± 6.7	84.7 ± 13.1	73.2 ± 9.6	75.2 ± 14.0	76.7 ± 12.5	72.9 ± 10.0	72.7 ± 12.2	71.6 ± 13.6	65.7 ± 10.5	71.4 ± 23.9
BMI (kg/m^2)	25.6 ± 3.9	22.5 ± 1.6	25.0 ± 3.4	31.8 ± 5.4	27.1 ± 4.8	26.5 ± 5.1	29.0 ± 5.3	27.3 ± 4.4	25.7 ± 3.8	27.3 ± 3.9	26.1 ± 3.8	26.9 ± 10.3
PROMIS physical function score	54.2 ± 7.1	62.0 ± 6.9	63.0 ± 4.4	52.2 ± 3.3	51.3 ± 3.8	57.9 ± 8.9	53.1 ± 6.9	51.4 ± 5.0	64.9 ± 5.7	48.3 ± 2.5	53.1 ± 4.0	54.6 ± 3.82
SF36-physical function score	96 ± 4	100 ± 0	100 ± 0	96 ± 4	93 ± 8	96 ± 5	92 ± 8	87 ± 6	99 ± 2	87 ± 12	85 ± 9	95 ± 6
Grip strength (kg)	17.4 ± 3.1	28.2 ± 0.9	37.0 ± 2.5	16.9 ± 6.5	26.8 ± 0.6	37.1 ± 2.6	16.7 ± 2.4	25.4 ± 0.3	35.8 ± 2.1	17.1 ± 4.0	21.5 ± 0.8	31.2 ± 3.0
Grip strength (z-score)	-1.72 ± 0.52	0.04 ± 0.13	1.43 ± 0.35	-1.73 ± 1.17	0.10 ± 0.15	1.90 ± 0.39	-1.53 ± 0.56	0.16 ± 0.13	1.98 ± 0.41	-0.74 ± 1.24	0.09 ± 0.16	1.91 ± 0.67
Repeat chair stands in 30s (n)	12.4 ± 2.2	17.8 ± 1.3	24.6 ± 3.1	11.4 ± 0.6	16.8 ± 0.8	25.6 ± 3.8	11.8 ± 0.8	15.0 ± 1.0	22.4 ± 2.1	11.4 ± 2.1	13.2 ± 1.3	19.8 ± 1.8
Repeat chair stands in 30s (z-score)	-1.55 ± 0.81	0.01 ± 0.37	1.53 ± 0.51	-1.56 ± 0.24	0.11 ± 0.21	1.87 ± 0.61	-1.16 ± 0.24	-0.10 ± 0.27	1.50 ± 0.42	-0.67 ± 0.65	-0.06 ± 0.49	1.55 ± 0.48
	MALES											
	20-35 years			35-50 years			50-65 years			65+		
	Low	Average	High	Low	Average	High	Low	Average	High	Low	Average	High
Age (yr)	23.9 ± 4.5	26.4 ± 3.1	26.9 ± 4.6	40.6 ± 2.2	40.9 ± 3.8	42.7 ± 5.5	62.2 ± 3.1	57.9 ± 5.5	54.1 ± 3.8	73.9 ± 6.6	70.5 ± 6.3	69.0 ± 5.5
Height (m)	1.77 ± 0.08	1.80 ± 0.06	1.82 ± 0.06	1.82 ± 0.05	1.74 ± 0.05	1.77 ± 0.07	1.77 ± 0.10	1.80 ± 0.05	1.79 ± 0.11	1.70 ± 0.04	1.74 ± 0.05	1.75 ± 0.04
Weight (kg)	71.0 ± 9.0	90.5 ± 15.0	82.8 ± 10.1	84.9 ± 11.8	87.9 ± 14.5	84.1 ± 10.3	80.1 ± 3.5	91.1 ± 13.5	87.7 ± 12.9	88.7 ± 14.8	80.3 ± 9.8	82.1 ± 8.4
BMI (kg/m^2)	22.9 ± 2.0	27.9 ± 4.6	24.9 ± 2.8	25.5 ± 3.3	29.1 ± 4.9	26.8 ± 3.1	26.8 ± 2.6	28.0 ± 3.2	27.4 ± 2.3	30.8 ± 6.5	26.5 ± 3.4	26.8 ± 3.1
PROMIS physical function score	56.2 ± 6.7	59.9 ± 5.6	66.2 ± 4.7	56.1 ± 9.5	58.1 ± 4.2	67.8 ± 1.5	54.3 ± 7.0	53.1 ± 1.3	58.6 ± 5.4	47.7 ± 6.3	55.7 ± 5.4	56.2 ± 6.4
SF36-physical function score	97 ± 4	99 ± 2	100 ± 0	95 ± 5	99 ± 6	100 ± 0	92 ± 8	97 ± 3	98 ± 3	51 ± 29	87 ± 19	93 ± 10
Grip strength (kg)	34.5 ± 3.7	47.8 ± 1.3	59.0 ± 3.2	40.3 ± 2.1	48.1 ± 1.2	56.5 ± 3.6	34.6 ± 3.8	43.1 ± 3.1	52.6 ± 6.0	23.0 ± 7.9	36.5 ± 1.4	41.8 ± 1.8
Grip strength (z-score)	-1.26 ± 0.34	0.03 ± 0.18	1.03 ± 0.24	-0.72 ± 0.22	0.06 ± 0.33	0.88 ± 0.27	-0.58 ± 0.51	0.21 ± 0.38	1.05 ± 0.67	-1.49 ± 0.95	0.13 ± 0.55	0.73 ± 0.24
Repeat chair stands in 30s (n)	12.0 ± 1.9	18.2 ± 1.5	26.6 ± 3.9	15.4 ± 0.9	19.2 ± 3.4	26.0 ± 3.8	13.4 ± 2.9	16.8 ± 1.1	21.8 ± 2.9	9.8 ± 1.9	14.8 ± 2.5	21.6 ± 2.3
Repeat chair stands in 30s (z-score)	-1.79 ± 0.72	0.04 ± 0.36	1.72 ± 0.61	-0.55 ± 0.22	0.33 ± 0.66	1.62 ± 0.62	-0.63 ± 0.80	0.14 ± 0.27	1.06 ± 0.49	-1.26 ± 0.63	0.02 ± 0.62	1.38 ± 0.56

Table S2. Characteristics on performance (high, average, low) groups, gender (female, male) and by age with gender group.

2.1 High performance group

Label	N	Mean	SD	Median	Min	Max
Age (years)	40	48.13	15.88	50.08	20.25	78.07
Height (cm)	40	172.59	8.68	170.70	158.60	191.80
Weight (kg)	40	78.14	13.65	75.90	54.10	113.50
BMI (kg/m^2)	40	26.25	4.52	25.20	19.70	45.10
Appendicular lean/height^2 (kg/m^2)	39	8.20	1.33	8.25	5.10	10.94
Total BMD (g/cm^2)	38	1.15	0.15	1.16	0.77	1.43
Spine BMD (g/cm^2)	39	1.09	0.17	1.12	0.68	1.40
Femoral Neck BMD (g/cm^2)	39	0.91	0.16	0.90	0.61	1.30

Total SPPB score	40	12.00	0.00	12.00	12.00	12.00
SPPB gait speed score	40	4.00	0.00	4.00	4.00	4.00
Usual gait speed (m/s)	40	1.45	0.18	1.46	1.08	2.00
Fast gait speed (m/s)	40	2.26	0.30	2.26	1.72	3.28
Best Grip Strength (kg)	40	43.86	10.49	39.80	28.30	63.00
Time for 5 chair stands (s)	40	6.21	1.12	6.27	2.13	7.84
6-min walk distance (m)	39	627.49	92.98	632.00	427.00	969.00
PROMIS score	40	61.14	6.83	60.90	48.50	73.30
SF-36 PFS raw score	40	97.63	5.06	100.00	75.00	100.00

2.2 Average

	N	Mean	SD	Median	Min	Max
Age (years)	40	50.03	17.53	49.59	22.84	81.14
Height (cm)	40	170.37	8.91	169.80	153.80	189.20
Weight (kg)	40	78.09	14.71	77.85	57.50	114.10
BMI (kg/m^2)	40	26.81	4.05	25.90	21.00	35.50
Appendicular lean/height^2 (kg/m^2)	40	7.81	1.54	7.80	5.12	12.14
Total BMD (g/cm^2)	40	1.10	0.14	1.11	0.84	1.38
Spine BMD (g/cm^2)	40	1.12	0.17	1.13	0.79	1.41
Femoral Neck BMD (g/cm^2)	40	0.90	0.18	0.86	0.60	1.35
Total SPPB score	40	11.85	0.58	12.00	9.00	12.00
SPPB gait speed score	40	4.00	0.00	4.00	4.00	4.00
Usual gait speed (m/s)	40	1.41	0.17	1.44	1.10	1.66
Fast gait speed (m/s)	40	2.05	0.27	2.06	1.52	2.74
Best Grip Strength (kg)	40	34.69	10.21	32.20	20.90	51.50
Time for 5 chair stands (s)	40	8.55	1.54	8.25	5.28	12.38
6-min walk distance (m)	39	561.82	80.40	566.00	367.00	700.00
PROMIS score	40	55.36	5.72	54.70	47.70	73.30
SF-36 PFS raw score	40	93.00	9.66	95.00	55.00	100.00

2.3 Low

	N	Mean	SD	Median	Min	Max
Age (years)	40	50.64	19.49	49.96	20.09	84.73
Height (cm)	40	169.13	9.03	167.25	149.00	187.10
Weight (kg)	40	78.16	12.77	79.40	58.10	113.70
BMI (kg/m^2)	40	27.44	4.86	26.30	20.50	41.50
Appendicular lean/height^2 (kg/m^2)	38	7.52	1.24	7.60	4.64	10.05
Total BMD (g/cm^2)	38	1.07	0.14	1.08	0.83	1.43
Spine BMD (g/cm^2)	38	1.06	0.17	1.04	0.74	1.45
Femoral Neck BMD (g/cm^2)	38	0.84	0.15	0.83	0.55	1.19
Total SPPB score	40	10.83	2.15	12.00	0.00	12.00
SPPB gait speed score	40	3.98	0.16	4.00	3.00	4.00
Usual gait speed (m/s)	40	1.32	0.18	1.33	0.74	1.89
Fast gait speed (m/s)	40	1.83	0.31	1.86	1.01	2.45
Best Grip Strength (kg)	40	25.05	10.19	21.80	6.60	43.00
Time for 5 chair stands (s)	40	11.86	2.56	11.23	7.39	19.77
6-min walk distance (m)	39	519.54	125.01	540.00	160.00	872.00
PROMIS score	40	52.75	6.65	51.60	40.40	68.80
SF-36 PFS raw score	40	88.25	18.31	95.00	20.00	100.00

2.4 Female

	N	Mean	SD	Median	Min	Max
Age (years)	60	50.25	17.34	50.10	20.25	84.30
Height (cm)	60	164.38	5.75	164.70	149.00	178.50
Weight (kg)	60	71.98	12.59	67.05	54.10	113.50
BMI (kg/m^2)	60	26.73	5.05	25.15	19.70	45.10
Appendicular lean/height^2 (kg/m^2)	57	6.97	1.06	6.95	4.64	9.03
Total BMD (g/cm^2)	56	1.05	0.14	1.05	0.77	1.35
Spine BMD (g/cm^2)	57	1.09	0.17	1.09	0.68	1.43
Femoral Neck BMD (g/cm^2)	57	0.87	0.16	0.84	0.60	1.26
Total SPPB score	60	11.67	0.68	12.00	9.00	12.00
SPPB gait speed score	60	4.00	0.00	4.00	4.00	4.00
Usual gait speed (m/s)	60	1.38	0.16	1.38	1.10	1.78
Fast gait speed (m/s)	60	1.97	0.28	1.90	1.41	2.55
Best Grip Strength (kg)	60	25.91	8.22	25.95	6.60	39.90
Time for 5 chair stands (s)	60	9.08	2.89	8.79	2.13	16.01
6-min walk distance (m)	59	543.22	80.51	555.00	348.00	713.00
PROMIS score	60	55.50	7.11	54.70	44.50	73.30
SF-36 PFS raw score	60	93.83	7.67	95.00	70.00	100.00

2.5 Male

	N	Mean	SD	Median	Min	Max
Age (years)	60	48.95	17.94	49.65	20.09	84.73
Height (cm)	60	177.01	6.79	177.00	165.10	191.80
Weight (kg)	60	84.28	11.77	83.70	61.60	114.10
BMI (kg/m^2)	60	26.94	3.87	26.05	20.50	41.50
Appendicular lean/height^2 (kg/m^2)	60	8.67	1.15	8.67	6.45	12.14
Total BMD (g/cm^2)	60	1.16	0.14	1.15	0.89	1.43
Spine BMD (g/cm^2)	60	1.10	0.16	1.09	0.74	1.45
Femoral Neck BMD (g/cm^2)	60	0.90	0.17	0.87	0.55	1.35
Total SPPB score	60	11.45	1.83	12.00	0.00	12.00
SPPB gait speed score	60	3.98	0.13	4.00	3.00	4.00
Usual gait speed (m/s)	60	1.41	0.20	1.43	0.74	2.00
Fast gait speed (m/s)	60	2.13	0.38	2.14	1.01	3.28
Best Grip Strength (kg)	60	43.15	10.57	43.20	10.80	63.00
Time for 5 chair stands (s)	60	8.66	3.03	7.82	4.42	19.77
6-min walk distance (m)	58	596.47	128.41	613.00	160.00	969.00
PROMIS score	60	57.33	7.38	56.20	40.40	70.30
SF-36 PFS raw score	60	92.08	16.42	100.00	20.00	100.00

2.6 By gender and age group

Gender	Age	Measurement	N	Mean	SD	Median	Min	Max
Female	20-34	Age (years)	15	27.18	4.11	26.78	20.25	34.68
		Height (cm)	15	165.48	5.74	165.80	156.00	173.50
		Weight (kg)	15	66.53	7.60	64.10	58.50	79.50
		BMI (kg/m^2)	15	24.37	3.20	23.20	21.00	32.00
		Appendicular lean/height^2 (kg/m^2)	15	7.12	1.07	7.06	5.10	8.83
		Total BMD (g/cm^2)	15	1.07	0.15	1.04	0.86	1.35
		Spine BMD (g/cm^2)	15	1.10	0.14	1.09	0.84	1.34
		Femoral Neck BMD (g/cm^2)	15	0.99	0.16	0.99	0.78	1.26
		Total SPPB score	15	11.73	0.59	12.00	10.00	12.00
		SPPB gait speed score	15	4.00	0.00	4.00	4.00	4.00
		Usual gait speed (m/s)	15	1.47	0.13	1.47	1.28	1.75
		Fast gait speed (m/s)	15	2.09	0.26	2.09	1.52	2.52
		Best Grip Strength (kg)	15	27.52	8.56	28.20	13.40	39.70
		Time for 5 chair stands (s)	15	8.70	3.20	7.57	4.95	16.01
		6-min walk distance (m)	15	568.00	87.06	566.00	348.00	700.00
		PROMIS score	15	59.75	7.09	60.40	44.50	73.30
		SF-36 PFS raw score	15	98.67	2.97	100.00	90.00	100.00
	35-49	Age (years)	15	44.84	4.45	46.81	35.04	49.05
		Height (cm)	15	165.66	5.47	166.60	155.80	175.50
		Weight (kg)	15	77.72	12.58	81.00	58.20	97.00
		BMI (kg/m^2)	15	28.48	5.35	29.50	20.40	37.20
		Appendicular lean/height^2 (kg/m^2)	13	7.36	0.92	7.52	5.66	9.03
		Total BMD (g/cm^2)	13	1.11	0.12	1.16	0.91	1.26
		Spine BMD (g/cm^2)	13	1.23	0.12	1.21	0.96	1.43
		Femoral Neck BMD (g/cm^2)	13	0.94	0.14	0.92	0.75	1.19
		Total SPPB score	15	11.67	0.62	12.00	10.00	12.00
		SPPB gait speed score	15	4.00	0.00	4.00	4.00	4.00
		Usual gait speed (m/s)	15	1.34	0.22	1.28	1.10	1.78
		Fast gait speed (m/s)	15	1.97	0.28	1.93	1.52	2.52
		Best Grip Strength (kg)	15	26.91	9.35	26.60	6.60	39.90
		Time for 5 chair stands (s)	15	8.55	3.24	7.95	2.13	13.79
		6-min walk distance (m)	15	546.80	96.45	510.00	394.00	713.00
		PROMIS score	15	53.79	6.25	52.10	48.20	73.30
		SF-36 PFS raw score	15	95.00	5.98	95.00	80.00	100.00
	50-64	Age (years)	15	56.13	3.53	55.95	51.16	62.61
		Height (cm)	15	164.92	5.77	163.30	155.50	178.50
		Weight (kg)	15	74.09	10.93	69.70	59.90	90.90

		BMI (kg/m^2)	15	27.33	4.42	26.70	20.90	35.50
		Appendicular lean/height^2 (kg/m^2)	15	6.93	1.26	6.77	4.64	8.98
		Total BMD (g/cm^2)	15	1.07	0.11	1.10	0.86	1.22
		Spine BMD (g/cm^2)	15	1.01	0.16	0.99	0.79	1.38
		Femoral Neck BMD (g/cm^2)	15	0.83	0.14	0.80	0.63	1.19
		Total SPPB score	15	11.87	0.35	12.00	11.00	12.00
		SPPB gait speed score	15	4.00	0.00	4.00	4.00	4.00
		Usual gait speed (m/s)	15	1.34	0.15	1.33	1.15	1.64
		Fast gait speed (m/s)	15	1.99	0.32	1.90	1.41	2.55
		Best Grip Strength (kg)	15	25.95	8.23	25.30	14.10	38.20
		Time for 5 chair stands (s)	15	9.17	2.30	9.44	5.02	12.06
		6-min walk distance (m)	14	549.71	69.86	561.50	412.00	658.00
		PROMIS score	15	56.47	8.29	54.70	47.70	73.30
		SF-36 PFS raw score	15	92.67	7.29	95.00	80.00	100.00
	65+	Age (years)	15	72.86	5.54	73.43	65.04	84.30
		Height (cm)	15	161.46	5.54	162.70	149.00	167.50
		Weight (kg)	15	69.58	15.96	63.80	54.10	113.50
		BMI (kg/m^2)	15	26.75	6.26	24.90	19.70	45.10
		Appendicular lean/height^2 (kg/m^2)	14	6.50	0.82	6.43	5.17	8.25
		Total BMD (g/cm^2)	13	0.96	0.13	0.93	0.77	1.24
		Spine BMD (g/cm^2)	14	1.03	0.18	1.02	0.68	1.33
		Femoral Neck BMD (g/cm^2)	14	0.73	0.08	0.74	0.60	0.87
		Total SPPB score	15	11.40	0.99	12.00	9.00	12.00
		SPPB gait speed score	15	4.00	0.00	4.00	4.00	4.00
		Usual gait speed (m/s)	15	1.37	0.13	1.36	1.11	1.58
		Fast gait speed (m/s)	15	1.82	0.19	1.78	1.54	2.27
		Best Grip Strength (kg)	15	23.27	6.69	21.20	10.70	35.70
		Time for 5 chair stands (s)	15	9.90	2.84	9.87	5.82	15.72
		6-min walk distance (m)	15	508.80	58.78	496.00	408.00	612.00
		PROMIS score	15	51.99	4.26	51.60	45.00	60.90
		SF-36 PFS raw score	15	89.00	9.86	90.00	70.00	100.00
Male	20-34	Age (years)	15	25.77	4.07	24.26	20.09	31.70
		Height (cm)	15	179.73	6.67	178.70	165.10	191.80
		Weight (kg)	15	81.45	13.61	77.40	61.60	111.10
		BMI (kg/m^2)	15	25.16	3.78	24.40	20.50	35.50
		Appendicular lean/height^2 (kg/m^2)	15	8.78	1.23	9.15	6.45	10.35
		Total BMD (g/cm^2)	15	1.20	0.09	1.18	1.08	1.43
		Spine BMD (g/cm^2)	15	1.12	0.15	1.15	0.83	1.34

		Femoral Neck BMD (g/cm^2)	15	1.06	0.21	1.07	0.63	1.35
		Total SPPB score	15	11.87	0.52	12.00	10.00	12.00
		SPPB gait speed score	15	4.00	0.00	4.00	4.00	4.00
		Usual gait speed (m/s)	15	1.41	0.18	1.42	1.10	1.68
		Fast gait speed (m/s)	15	2.22	0.43	2.17	1.42	3.28
		Best Grip Strength (kg)	15	47.09	10.73	46.70	30.30	63.00
		Time for 5 chair stands (s)	15	8.45	2.83	8.15	4.42	15.72
		6-min walk distance (m)	15	659.60	138.25	665.00	296.00	969.00
		PROMIS score	15	60.74	6.82	59.70	50.00	70.30
		SF-36 PFS raw score	15	98.67	2.97	100.00	90.00	100.00
	35-49	Age (years)	15	40.86	3.62	39.17	35.59	48.18
		Height (cm)	15	177.76	6.52	178.80	166.50	187.50
		Weight (kg)	15	85.64	11.53	85.30	66.80	105.70
		BMI (kg/m^2)	15	27.15	3.88	26.10	20.90	35.20
		Appendicular lean/height^2 (kg/m^2)	15	8.72	1.44	8.64	6.55	12.14
		Total BMD (g/cm^2)	15	1.22	0.15	1.28	0.95	1.40
		Spine BMD (g/cm^2)	15	1.04	0.15	1.08	0.74	1.41
		Femoral Neck BMD (g/cm^2)	15	0.87	0.10	0.89	0.70	1.05
		Total SPPB score	15	12.00	0.00	12.00	12.00	12.00
		SPPB gait speed score	15	4.00	0.00	4.00	4.00	4.00
		Usual gait speed (m/s)	15	1.51	0.24	1.49	1.08	2.00
		Fast gait speed (m/s)	15	2.23	0.27	2.27	1.67	2.67
		Best Grip Strength (kg)	15	48.33	7.37	48.50	37.20	61.40
		Time for 5 chair stands (s)	15	7.25	1.61	7.74	4.51	9.35
		6-min walk distance (m)	15	615.67	95.42	616.00	427.00	872.00
		PROMIS score	15	60.09	7.95	60.70	44.90	70.30
		SF-36 PFS raw score	15	97.00	4.93	100.00	85.00	100.00
	50-64	Age (years)	15	58.03	5.23	59.40	51.12	64.96
		Height (cm)	15	177.39	7.87	178.00	165.40	188.10
		Weight (kg)	15	86.31	11.21	84.20	69.40	114.10
		BMI (kg/m^2)	15	27.39	2.56	26.50	24.30	33.20
		Appendicular lean/height^2 (kg/m^2)	15	8.75	1.04	8.56	6.77	10.94
		Total BMD (g/cm^2)	15	1.13	0.15	1.13	0.89	1.43
		Spine BMD (g/cm^2)	15	1.13	0.14	1.10	0.94	1.40
		Femoral Neck BMD (g/cm^2)	15	0.86	0.09	0.85	0.70	1.01
		Total SPPB score	15	11.67	0.90	12.00	9.00	12.00
		SPPB gait speed score	15	4.00	0.00	4.00	4.00	4.00
		Usual gait speed (m/s)	15	1.40	0.13	1.40	1.24	1.60

		Fast gait speed (m/s)	15	2.15	0.30	2.06	1.77	2.74
		Best Grip Strength (kg)	15	43.42	8.66	43.90	28.80	62.00
		Time for 5 chair stands (s)	15	8.83	2.47	7.96	5.37	15.73
		6-min walk distance (m)	14	597.00	62.60	598.00	447.00	700.00
		PROMIS score	15	55.31	5.36	54.00	47.00	64.30
		SF-36 PFS raw score	15	95.67	5.63	95.00	80.00	100.00
	65+	Age (years)	15	71.14	6.09	70.10	65.19	84.73
		Height (cm)	15	173.17	4.54	173.70	165.60	180.80
		Weight (kg)	15	83.71	11.16	82.60	67.30	113.70
		BMI (kg/m^2)	15	28.04	4.72	27.60	21.80	41.50
		Appendicular lean/height^2 (kg/m^2)	15	8.44	0.90	8.16	7.16	10.05
		Total BMD (g/cm^2)	15	1.08	0.12	1.05	0.91	1.35
		Spine BMD (g/cm^2)	15	1.09	0.21	1.07	0.77	1.45
		Femoral Neck BMD (g/cm^2)	15	0.79	0.11	0.79	0.55	0.98
		Total SPPB score	15	10.27	3.31	12.00	0.00	12.00
		SPPB gait speed score	15	3.93	0.26	4.00	3.00	4.00
		Usual gait speed (m/s)	15	1.33	0.23	1.36	0.74	1.63
		Fast gait speed (m/s)	15	1.90	0.42	2.06	1.01	2.35
		Best Grip Strength (kg)	15	33.75	9.29	36.60	10.80	43.80
		Time for 5 chair stands (s)	15	10.12	4.19	7.80	6.13	19.77
		6-min walk distance (m)	14	507.71	156.49	545.50	160.00	703.00
		PROMIS score	15	53.19	6.89	54.00	40.40	66.10
		SF-36 PFS raw score	15	77.00	27.24	85.00	20.00	100.00

Table S3a. Correlations of D-BAIBA with age and characteristics of physical performance in 120 individuals (aged= 20–85 years).

			D-BAIBA			
			Pearson		Spearman	
Gender	Parameters	N	P	p-value	S	p-value
Both	Age	120	0.1961	0.0319		
	Height	120				
	Usual gait speed	120			0.1983	0.0299
	Fast gait speed	120				

	Six Min Distance	117		
	Total SPPB score	120		
	PROMIS score	120		
	SF36 PFS raw score	120		
	Subtotal BMD	117		
	Femoral neck BMD	117		
Female	Age	60		
	SF36 PFS raw score	60		
	Femoral neck BMD	57		
Male	Age	60		
	BMI	60		
	Height	60		
	Usual gait speed	60		
	Six Min Distance	58	0.2734	0.0379
	PROMIS score	60		
	SF36 PFS raw score	60		
	Subtotal BMD	60		
	Femoral neck BMD	60		

Pearson correlations (P) and Spearman Rank correlations (S) with p-value < 0.05 are shown in the Table.

Table S3b. Correlations of L-BAIBA with age and characteristics of physical performance in 120 individuals (aged 20–85 years).

		120 participants		
		N	Spearman	
Gender	Variables		S	p-value
Both	Age			
	Weight	120		
	BMI	120	0.22851	0.01206
	Total fat mass	117	0.18651	0.04407
	Subtotal BMD	117		
Female	Age			
	Weight	60	0.27028	0.03674
	BMI	60	0.26381	0.04168
	Total SPPB score	60		
	SF36 PFS raw score			
	Subtotal BMD	57	0.27988	0.03498
Male	Usual gait speed			
	Total fat mass	60	0.25811	0.04646
	Total % fat mass	60		

Spearman Rank correlations (S) with p-value < 0.05 are shown in the Table.

Table S4a. L-BAIBA in ♀ using Spearman correlation ($p < 0.05$)

			**LBAIBA**					
			**Ordinary**		**Partial on age**		**Partial on age, BMI**	
	**group**	**N**	**correlation**	**p value**	**correlation**	**p value**	**correlation**	**p value**
BMI	Overall	60	0.26	0.0417				
	Average	20	0.49	0.0274				
Total BMD	Overall	56	0.28	0.0358	0.29	0.0287		
Subtotal BMD	Overall	57	0.28	0.0350	0.32	0.0164		
Femoral Neck BMD	High	19	0.50	0.0285			0.48	0.0498
Total Lean Mass	High	19	0.47	0.0407				
Total Fat Percent	High	19					-0.53	0.0275
	Average	20	0.50	0.0243				
Appendicular lean/height^2(kg/m^2)	Low	18	-0.51	0.0291			-0.52	0.0404

Table S4b. L-BAIBA in ♂ using Spearman correlation (p <0.05)

Variable	group	N	LBAIBA					
			Ordinary		Partial on age		Partial on age, BMI	
			correlation	p value	correlation	p value	correlation	p value
SixMinDistance	Low	20			0.53	0.0199	0.53	0.0279
Femoral Neck BMD	High	20	-0.46	0.0430	-0.48	0.0372		
	Average	20	-0.47	0.0387				
Total Fat Mass	Overall	60	0.26	0.0465				

Table S5. D-BAIBA in **overall**, **LP** and **HP** ♂ using Pearson and Spearman correlation.

Variable	group	N	DBAIBA (Pearson)						DBAIBA (Spearman)					
			Ordinary		Partial on age		Partial on age, BMI		Ordinary		Partial on age		Partial on age, BMI	
			correlation	p value	correlation	p value	correlation	p value	correlation	p value	correlation	p value	correlation	p value
Usual gait speed	Overall	60									0.27	0.04	0.26	0.049
Six Min Distance	Overall	58	0.27	0.04	0.37	0.00	0.34	0.0090						
Femoral Neck BMD	Overall	60			0.30	0.02	0.32	0.0130			0.32	0.01	0.33	0.01
Six Min Distance	LP	20	0.54	0.01	0.56	0.01	0.56	0.01	0.47	0.04	0.46	0.05		
Appendicular lean/height^2 (kg/m^2)	HP	20							-0.52	0.02	-0.49	0.03		

Table S6. D-BAIBA in the **LP** ♀ using Pearson and Spearman correlation

Variable	group	N	DBAIBA (Pearson)						DBAIBA (Spearman)				
			Ordinary		Partial on age		Partial on age, BMI		Ordinary		Partial on age		Partia
			correlation	p value	correlation	p value	correlation	p value	correlation	p value	correlation	p value	correlation
Age	LP	20	0.49	0.03									
Best Grip Strength	LP	20					0.52	0.03	0.45	0.04	0.48	0.04	0.49
Time for 5 chair stands	LP	20							0.49	0.03	0.52	0.02	0.52
RCS30s	LP	20	-0.47	0.04					-0.51	0.02	-0.47	0.04	
Femoral NeckBMD	LP	18	-0.52	0.03					-0.63	0.01			

Table S7. D-BAIBA in ♂ using Pearson correlation.

Variable	group	N	DBAIBA (Pearson correlation)					
			Ordinary		Partial on age		Partial on age, BMI	
			correlation	p value	correlation	p value	correlation	p value
Six Min Distance	Overall	58	0.27338	0.0379	0.37319	0.0042	0.34598	0.0090
	High	19					0.50339	0.0394
	Low	20	0.53931	0.0141	0.56149	0.0124	0.56395	0.0148
Femoral Neck BMD	Overall	60			0.29682	0.0224	0.32446	0.0130
Total Lean Mass	Overall	60					0.28114	0.0325
Total Fat Mass	High	20			-0.52328	0.0215		
Total Fat Percent	Overall	60			-0.26788	0.0402		
	High	20			-0.51767	0.0232		

Table S8. D-BAIBA in ♂ using Spearman correlation.

Variable	group	N	DBAIBA (Spearman correlation)					
			Ordinary		Partial on age		Partial on age, BMI	
			correlation	p value	correlation	p value	correlation	p value
Usual gait speed	Overall	60			0.27203	0.0371	0.25891	0.0497
RCS_30s	High	20					0.47116	0.0484
Six Min Distance	Low	20	0.46917	0.0369	0.45984	0.0476		
Femoral Neck BMD	Overall	60			0.32240	0.0128	0.33456	0.0103
	Average	20			0.53998	0.0170	0.56890	0.0137
Appendicular lean/height^2(kg/m^2)	High	20	-0.51729	0.0195	-0.48907	0.0336		

Table S9. D-BAIBA in LP ♀ using Pearson correlation with age; and age & BMI effect removed.

Variable	group	N	DBAIBA (Pearson correlation)					
			Ordinary		Partial on age		Partial on age, BMI	
			correlation	p value	correlation	p value	correlation	p value
Age	Low	20	0.49099	0.0279				
Best Grip Strength	Low	20					0.51806	0.0276
Time for 5 chair stands	Low	20						
RCS_30s	Low	20	-0.47192	0.0357				
Femoral Neck BMD	Low	18	-0.51533	0.0286				

Table S10. D-BAIBA in LP ♀ using Spearman correlation with age; and age & BMI effect removed.

			DBAIBA (Spearman correlation)					
			Ordinary		Partial on age		Partial on age, BMI	
Variable	group	N	correlation	p value	correlation	p value	correlation	p value
Best Grip Strength	Low	20	0.45424	0.0442	0.48078	0.0372	0.49167	0.0382
Time for 5 chair stands	Low	20	0.49323	0.0271	0.51900	0.0228	0.51811	0.0276
RCS_30s	Low	20	-0.51398	0.0204	-0.46774	0.0434		
Femoral Neck BMD	Low	18	-0.63055	0.0050				
